# Concurrent Hospice and Dialysis Care: Considerations for Implementation

**DOI:** 10.1007/s11606-023-08504-w

**Published:** 2023-11-14

**Authors:** Natalie C. Ernecoff, Mayumi T. Robinson, Erica M. Motter, Alexandra E. Bursic, Keith Lagnese, Robert Taylor, Dale Lupu, Jane O. Schell

**Affiliations:** 1 RAND Corporation, 4570 Fifth Avenue, #600, Pittsburgh, PA 15213 USA; 2 Division of General Internal Medicine, University of Pittsburgh School of Medicine, Pittsburgh, PA USA; 3 Division of Renal-Electrolyte, University of Pittsburgh School of Medicine, Pittsburgh, PA USA; 4 Optum Home & Community Care, Landmark Health, Huntington Beach, CA USA; 5 Dialysis Clinic, Inc, Nashville, TN USA; 6 School of Nursing, George Washington University, Washington, DC USA

## Abstract

**Importance:**

Hospice positively impacts care at the end of life for patients and their families. However, compared to the general Medicare population, patients on dialysis are half as likely to receive hospice. Concurrent hospice and dialysis care offers an opportunity to improve care for people living with end-stage kidney disease (ESKD).

**Objective:**

We sought to (1) develop a conceptual model of the Program and (2) identify key components, resources, and considerations for further implementation.

**Design:**

We conducted a template analysis of qualitative interviews and convened a community advisory panel (CAP) to get feedback on current concurrent care design and considerations for dissemination and implementation.

**Participants:**

Thirty-nine patients with late-stage chronic kidney disease (CKD), family caregivers, bereaved family caregivers, hospice clinicians, nephrology clinicians, administrators, and policy experts participated in interviews. A purposive subset of 19 interviewees composed the CAP.

**Main Measures:**

Qualitative feedback on concurrent care design refinements, implementation, and resources.

**Key Results:**

Participants identified four themes that define an effective model of concurrent hospice and dialysis: it requires (1) timely goals-of-care conversations and (2) an interdisciplinary approach; (3) clear guidelines ensure smooth transitions for patients and families; and (4) hospice payment policy must support concurrent care. CAP participants provided feedback on the phases of an effective model of concurrent hospice and dialysis, and resources, including written and interactive educational materials, communication tools, workflow processes, and order sets.

**Conclusions:**

We developed a conceptual model for concurrent hospice and dialysis care and a corresponding resource list. In addition to policy changes, clinical implementation and educational resources can facilitate scalable and equitable dissemination of concurrent care. Concurrent hospice and dialysis care must be systematically evaluated via a hybrid implementation-effectiveness trial that includes the resources outlined herein, based on our conceptual model of concurrent care delivery.

## INTRODUCTION

Over 500,000 Americans are living with end-stage kidney disease (ESKD) on dialysis.^1^ For many people, dialysis confers a high mortality and morbidity, especially at end of life. Most patients are hospitalized in the last month of life with nearly half receiving intensive care.^2–6^ Hospice is associated with improved end-of-life outcomes, demonstrating favorable impact on quality of life and symptom burden, patient and caregiver satisfaction, and quality of care.^7–10^ Yet compared to patients with other end-stage diagnoses, people receiving dialysis are half as likely to receive hospice, and of those who do, nearly half spend 3 or fewer days on hospice before death.^11, 12^

The current structure of the Medicare Hospice Benefit poses a barrier to timely hospice enrollment for patients with ESKD in the USA. Hospices are required to cover the costs of treatments related to the terminal conditions, such as dialysis for people with ESKD. Therefore, hospices rarely accept patients who wish to continue such expensive treatments.^12–15^ This effectively forces patients to discontinue dialysis to receive hospice services, even if continuing dialysis may support their goals.^19^ Therefore, to provide goal-concordant care for people living with ESKD, policy and care models are needed to provide comfort-focused care without requiring discontinuation of dialysis.

We previously piloted a Concurrent Hospice-Dialysis Program (“the Program”), in which non-profit hospice and dialysis organizations collaborated to provide palliative (i.e., comfort-focused) dialysis treatments to patients after hospice enrollment. The Program was associated with a median hospice length-of-stay of 9 days among participants (13 days among those who received at least one dialysis treatment, 6 days longer than people with ESKD generally have hospice), and no participants died in the hospital.^16, 17^ Half of Program enrollees did not receive any dialysis treatments upon enrollment in hospice. Qualitative interviews with bereaved family caregivers and clinicians found that the Program facilitated goal-concordant care and provided a bridge to hospice to ease distress.^18^ Interviewees underscored the necessity of close partnerships and clear pathways between nephrology and hospice clinicians to facilitate the delivery of concurrent care. Therefore, we sought to develop a conceptual model of the Program and identify key components, resources, and considerations for further implementation.

## METHODS

### Study Design

We (1) conducted qualitative interviews about design and considerations for dissemination and implementation of a Concurrent Hospice-Dialysis Program and (2) convened a quarterly Community Advisory Panel (CAP) to inform Program refinements and resources, and (3) defined and outlined implementation and resources based on interviewee and CAP feedback. All study procedures were approved by the University of Pittsburgh Institutional Review Board.

### Program Participants and Eligibility

Program eligibility and utilization characteristics are described elsewhere.^16^ Briefly, patients receiving dialysis are considered appropriate for the Program when (1) their clinical team estimates a prognosis < 2 months or (2) patients and/or families request the Program or indicate changes in goals of care. The Program was offered to patients whose goals aligned with comfort and the hospice diagnosis was deemed related to their ESKD. Patients were not required to discontinue dialysis if they survived longer than 2 months.

### Conceptual Model

Program implementation was based on the Exploration, Preparation, Implementation, Sustainment (EPIS) Framework, which highlights processes and factors within an organization and in the context of the larger system.^19^ This framework allows evaluation and refinement of the existing Program, while facilitating identification of external factors relevant to implementation and dissemination.^20^

### Interview and Community Advisory Panel (CAP) Participants and Eligibility

We recruited a purposive sample by email and conducted interviews with patients with stage 4–5 CKD, family caregivers, bereaved family caregivers, hospice and nephrology clinicians (physicians, advanced practice providers, nurses, social workers) and administrators, and policy experts (national hospice policy organizations, health plans). Patients and family caregivers were from one health system; other participants were recruited nationally. Bereaved family caregivers had a loved one who was enrolled in the Program. Interviews were conducted from October 2020 to September 2021 via teleconference and lasted approximately 30 min. A subset of interviewees was invited to join the CAP which convened four times between 12/2020 and 10/2021. Participants received $30 for each interview and CAP session in which they participated.

### Data Collection

#### Quantitative

All participants provided demographic characteristics via electronic REDCap survey.

#### Qualitative

We conducted a qualitative analysis consistent with best practices.^21^ We developed an interview guide based on the EPIS Framework to elicit perceptions of the Program and operational aspects of the Program. We conducted semi-structured interviews until we reached thematic saturation across participant categories (i.e., patients, family caregivers, clinicians, administrators).^22^ Two trained investigators (EMM, MTR) conducted interviews with regular quality checks, debriefing, and acknowledgement of implicit bias (NCE, JOS). All interviews were conducted by telephone, audio-recorded, and transcribed.

### Community Advisory Panel (CAP)

We invited a purposeful, representative, and diverse subset of interview participants to join the CAP. Each CAP was held over Zoom for 60–90 min and video-recorded. CAPs included content overview and facilitated large-group discussions (JOS) and focused small-group breakout sessions (JOS, NCE, KL, DL) of 6–7 members. To maximize inclusion of diverse CAP feedback, we provided participants with multiple opportunities to comment on both CAP content and process, including post-meeting anonymous surveys and direct communication with study investigators.

**Figure 1:**
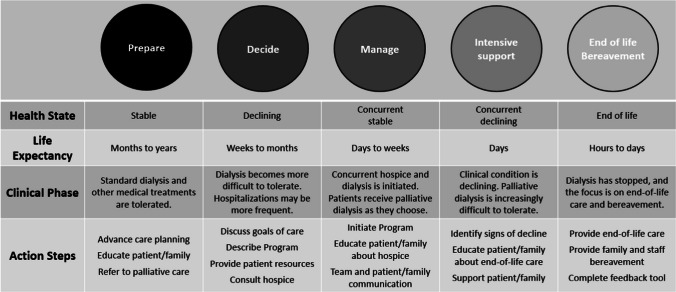
Conceptual model of Concurrent Hospice-Dialysis.

The first CAP (CAP 1) introduced the Program and elicited feedback on areas of strength and weakness in program design and implementation. CAP 2 characterized the Program’s current state, generating descriptions of a target state, and identifying specific steps to achieve the target state. To facilitate discussion, members were divided among three breakout groups organized by theme: Patient and Family Communication, Policy and Funding, and Hospice and Dialysis Workflow. CAP 3 introduced the five phases of the Concurrent Hospice-Dialysis Program Conceptual Model (Fig. 1), derived from themes generated from qualitative interviews and refined in CAP follow-up in sessions 1 and 2, and invited participants to identify needs (e.g., informational, administrative, financial) within each phase. CAP 4 presented components of educational and training materials relevant to the Program and elicited refinement and elaboration of resources for communication training; team education; patient and family education; workflow; documentation and care coordination; and team, patient, and family caregiver support.

### Data Analysis

#### Quantitative

Demographic characteristics of participants are described using univariable statistics (Excel).

#### Qualitative

We analyzed interviews using template analysis, an approach in which an initial a priori coding framework is iteratively modified throughout the coding process.^23, 24^ Two investigators (EMM, MTR) independently coded the same subset of transcripts line-by-line, and the framework was further refined by consensus (NCE, JOS). After a coding framework was established using the EPIS Framework, inter-rater reliability was assessed through administration of a structured test to both coders, consisting of passages from transcripts. Both coders passed the test when they independently applied codes with 95% agreement. One coder (EMM) then coded all remaining transcripts and met regularly with the second coder (MTR) to discuss. We used NVivo (QSR International) for qualitative data management.

To consolidate data from the CAP, we had a trained, dedicated notetaker for the full-group sessions and any breakout groups. After each CAP meeting, the investigator team and notetakers debrief, discussed key findings, specific pieces of feedback to be incorporated into the Program, and plans for future implementation. Findings were systematically documented, summarized, and developed into a conceptual model for delivery of concurrent hospice and dialysis, which was vetted in CAP 4. In addition, CAP members identified specific resources that would be helpful for patients, families, clinicians, and/or administrators; we also systematically summarized these resources.

## RESULTS

### Descriptive Characteristics of Interviewees and Community Advisory Panel

We interviewed 39 participants (Table 1): 2 patients, 1 patient-caregiver dyad, 4 bereaved family caregivers, 24 clinicians (9 physicians; 13 hospice/palliative care, 11 nephrology), and 8 administrators (4 hospice, 2 dialysis, and 2 policy experts). Interviewees were 39% male and 82% white.

**Table 1:** Demographic Characteristics of Interviewees and CAP Members

Characteristics	Interviewees, No. (%) (*n* = 39)^a^	CAP members, No. (%) (*n* = 19)
Age (years; mean ± SD)	52.1 ± 13.3	56.9 ± 14.5
Female	24 (62)	9 (47)
Race^b^		
White	32 (82)	15 (79)
Black	1 (3)	1 (5)
Asian	5 (13)	4 (21)
Not reported	1 (3)	1 (5)
Patients	2 (5)	2 (11)
Family caregivers	1 (3)	1 (5)
Bereaved family caregivers	4 (10)	2 (11)
Relationship to patient		
Spouse/partner	1 (20)	2 (67)
Child	3 (60)	1 (33)
Sibling	1 (20)	-
Hospice clinicians	7 (18)	3 (16)
APPs	1 (14)	-
Clinician fellows	1 (14)	-
Nurses	3 (43)	1 (33)
Nurse managers	2 (29)	2 (67)
Bereavement counselors	1 (14)	-
Dialysis clinicians	7 (18)	1 (5)
Social workers	4 (57)	1 (100)
Nurses	3 (43)	-
Nephrologists	4 (10)	3 (16)
Specialty palliative care	6 (15)	-
Physicians	5 (83)	-
Nurses	1 (17)	-
Years in practice among clinicians (mean ± SD)	16.2 ± 11.4	18.6 ± 14.2
Hospice administrators	4 (10)	3 (16)
MDs	2 (50)	2 (67)
Other	2 (50)	1 (33)
Dialysis administrators	2 (5)	2 (11)
MDs	1 (50)	2 (100)
Other	1 (50)	-
Policy experts	2 (5)	2 (11)
Years in position (mean ± SD)	7.5 ± 9.7	9.2 ± 10.8

^a^All percentages are rounded to the nearest whole number. Columns may not add to 100% because of rounding

^b^Survey respondents could select more than one race

Nineteen interviewees participated in the CAP (Table 1). The CAP was comprised of 26% patients and family caregivers, 37% nephrology and hospice clinicians, and 37% administrators (representing dialysis, hospice, and policy experts). Members were 53% male and 79% white.

### Qualitative Findings

Four themes, described below, emerged in our analysis. Table 2 includes quotes for all findings.

**Table 2:** Qualitative Results and Exemplar Quotes from One-on-One Interviews

**Theme 1: Effective concurrent care requires timely goals-of-care conversations**	
Conversations must start early and happen often	**Nephrologist**: I really wish [patients] would have those conversations more regularly so that their caregivers, their family members, and their clinicians are aware of their end-of-life wishes. And that would help us align their care when they reach that stage **Caregiver:** I would opt for earlier, because […] me and my mom have had these hard conversations. So, I knew what her choices were
Patients and caregivers need education and support to make informed decisions	**Patient:** I think it’s natural if you’re facing this condition, the best thing to do is know as much as you can about it. **Dialysis social worker**: The education and the psychosocial support leading up to the decision to withdraw or to stop dialysis is really important, and to help people communicate with their family what their wishes would be. **[**…] Also for families to know what to expect as someone is passing […] so that they know what might be coming and what’s normal or not
Physician discomfort hinders goals-of-care conversations	**Hospice administrator**: Doctors don’t always have the skills, knowledge, or experience to have that end-of-life conversation. So typically, you wait for some sort of event to occur that you can then say it is inevitable. So I think for physicians to be able to say, “When do you stop curing and start comforting,” and then really using either a palliative care or a hospice care consult to be able to take it from there and to help the family and the patient understand what their options are, help them prepare appropriately **Dialysis social worker**: It’s vitally important that the doctors become more comfortable with having these types of conversations with patients, because they’re definitely not
Goals-of-care conversations must include, but are not limited to, physician participation	**Dialysis social worker**: Definitely the physician. But I think the social worker plays a big role in that because they’re having those conversations with families and with patients on a day-to-day basis in the clinics **Hospice Administrator**: If the entire team is educated and well-versed on a program, anyone from the team could suggest. But […] definitely the physician has to be involved in having those initial conversations
**Theme 2: Effective concurrent care requires an interdisciplinary approach**	
Close collaboration and frequent communication between clinicians are essential	**Hospice administrator**: Frequent, open communication. Communication to lay the groundwork to build the program, but then daily or every other day or after the dialysis session **Nephrologist:** […] people in end-stage renal disease who are declining, they need support from both [dialysis and hospice] because they bring different kinds of expertise. And the interesting thing about Concurrent is its ability to sort of mesh those two worlds together
The whole team must have a common understanding of palliative dialysis	**Hospice administrator**: I think it’s important that the hospice team have education on providing care to a patient who’s actively receiving dialysis. Those of us who’ve been doing hospice care for the majority of our nursing career are not familiar with– not just hemodialysis, but peritoneal dialysis in a home and complications of that. **Dialysis nurse:** I think for staff, there needs to be education on hospice. Many people are still under the old concept that there’s no treatment. Once you enter hospice, everything stops, which is not necessarily true **Dialysis administrator**: The hospice staff has to be trained so that they do understand all the issues that are related to dialysis. For example, some of these patients might have a dialysis catheter. They should know about it
**Theme 3: Clear program guidelines ensure smooth transitions for patients and families**	
Programs must provide clear eligibility guidelines	**Dialysis social worker**: Bullet points for a team in a clinic to say, does this describe your patient? Have they had more hospitalizations? Have they had a functional decline? Would you be surprised if [this patient died]? **Hospice nurse**: It almost would need to be an algorithm: Do they have this diagnosis? Is there evidence of heart failure? Is there evidence of other—like COPD and hospitalizations, that would make it more of an identifiable patient that would be appropriate
Programs must clearly define roles	**Dialysis social worker**: Sometimes, clinics seem to trip over who’s making those treatment orders and treatment decisions. Is it the nephrologists at the clinic? Is it somebody from [hospice]? **Hospice administrator**: All the normal logistics, how many treatments, which dialysis companies are going to be involved, who are the support staff? Are we going to do most of the education? Are they going to do it? How do we work together between their caseworkers and social workers and ours? When does the handoff occur? My hope is that that’s pretty seamless, that we’re all working together for whatever that period of time is. And the first part of it clearly has to be with nephrologists because they’re still managing dialysis. But as we get closer to the hospice level of care that we seamlessly move into our team becoming the primary care team
Programs must clearly define palliative dialysis parameters	**Hospice nurse**: I think a barrier would be if a person was approved for so many dialysis visits and then they wanted to keep extending it. I think that’s where the problem’s going to be **Hospice nurse manager**: I prefer to have a more specified end to the dialysis to say to the family they’ll have this many treatments or this period of time. […] Sometimes when we leave those timelines open-ended, I think it causes more stress and anxiety for the family and patient
**Theme 4: Hospice payment policy must support concurrent care**	
Current Medicare hospice policy is a barrier to concurrent care	**Nephrologist:** There are some unique challenges for people on dialysis with regard to the way the Medicare Hospice Benefit is set up, how that disenfranchises patients who are on dialysis **Health plan administrator**: What I’ve heard from hospice providers is they can’t afford to do it. So they might do a dialysis treatment once or twice, but it’s very expensive and they’re not funded for it, so it’s a financial strain on the hospice to do dialysis care
Existing quality metrics disincentivize palliative dialysis	**Policy administrator**: [It would be helpful to measure] quality of life, patient satisfaction, families can weigh in, […] So what are all the quality measures, are we using the right ones **Palliative care physician**: How does the dialysis unit do this when they’re measured on so many things that these patients are going to screw up their measurements on? What’s their Kt/V? What’s their bicarb level? All of these things that Medicare measures to say whether you’re a good dialysis unit or not. And now we’re not going to look at any of those things, and how do I actually do that as a dialysis unit without having detrimental things happen to my unit?
Patient diversity must be factored into care delivery	**Palliative care physician**: Due to systemic factors, racism, economic discrimination, many of the patients may not have the resources to do hospice at home **Dialysis social worker**: I haven’t had any of my patients that do not speak English; […] I can imagine that it would create a big problem, because our own ability to communicate with these patients and families is limited **Palliative care nurse**: And then you get into like, ‘Are we being biased based on which demographic that we’re serving, based on which area that they’re close to, based on which unit that they’re close to?’
Evidence for concurrent care may drive policy reform	**Policy administrator**: I think it’s rallying a provider group, like the hospice and palliative care community around a demonstration, around maybe a supplement or an add-on to an existing model. […] If there’s momentum and a desire and a focus, it’s possible **Palliative care nurse**: I always think about this in terms of money, right, and how money talks. And I think that if you were to approach a large academic center and say, “We’ve done this with six patients,” I really, truly believe that you could reduce hospitalizations in a goal concordant way, […] and save hundreds and thousands of dollars for these patients who don’t want to be re-hospitalized but still want to continue dialysis

#### Theme 1: Effective Concurrent Care Requires Timely Goals-of-Care Conversations

Interviewees indicated that effective concurrent care requires timely goals-of-care conversations to support decision-making along the care continuum. Interviewees emphasized that conversations must start early and happen often to help families avoid uncertainty around the patient’s wishes at later stages of illness. One caregiver said, “I would opt for earlier, because […] me and my mom have had these hard conversations. So, I knew what her choices were.” A nephrologist noted that frequent goals-of-care conversations could provide clarity about the patient’s goals for both families and clinicians “[…] that would help us align their care when they reach that stage.”

Additionally, patients and caregivers need education and support to make informed decisions. One patient remarked, “I think it’s natural if you’re facing this condition, the best thing to do is know as much as you can about it.” Interviewees identified anticipatory guidance around end-of-life symptoms for patients with ESKD as especially important.

Interviewees noted that physician discomfort hinders goals-of-care conversations, which can lead to delayed conversations. Even still, interviewees emphasized that goals-of-care conversations must include, but are not limited to, physician participation. One hospice administrator stated, “I think that if the entire team is educated and well-versed on a program, then it would be nice if anyone from the team could suggest. But […] definitely the physician has to be involved in having those initial conversations.”

#### Theme 2: Effective Concurrent Care Requires an Interdisciplinary Approach

Close collaboration and communication between clinicians are essential and foster a unique co-management approach in the Program. One nephrologist observed, “[…] people in end-stage renal disease who are declining, they need support from both [dialysis and hospice] because they bring different kinds of expertise. And the interesting thing about Concurrent is its ability to sort of mesh those two worlds together.” A palliative care physician remarked that communication and care coordination across teams were facilitated by the interdisciplinary structure of the Program even prior to a patient joining the Program.

Interviewees representing both dialysis and hospice emphasized that the whole team must have a common understanding of palliative dialysis and each other’s roles and scope of practice. One hospice nurse said, “[…] understanding exactly what dialysis is doing, some people have a really good concept, and some people don’t.” In turn, dialysis clinicians cited the need for education on the hospice philosophy of care. One dialysis nurse said, “I think for staff, there needs to be education on hospice. Many people are still under the old concept that there’s no treatment. Once you enter hospice, everything stops, which is not necessarily true.”

#### Theme 3: Clear Program Guidelines Ensure Smooth Transitions for Patients and Families

Interviewees noted that Programs must provide clear eligibility guidelines and suggested standardized criteria, such as clinical algorithms or checklists, to aid identification of appropriate patients. Programs must clearly define the role of each discipline to prevent lapses in care coordination. As one palliative care physician described, “close collaboration between the nephrologist and the hospice. We don’t want these patients falling through the cracks where each side is saying, ‘Well, that’s not really my issue.’”.

Interviewees emphasized that Programs must clearly define palliative dialysis parameters to (1) limit the financial and logistical burdens of additional dialysis treatments and (2) prepare patients psychologically for stopping dialysis. A hospice nurse manager noted, “Sometimes when we leave those timelines open-ended, I think it causes more stress and anxiety for the family and patient.”

#### Theme 4: Hospice Payment Policy Must Support Concurrent Care

Interviewees described how current Medicare hospice eligibility and payment policy is a barrier to concurrent care. One nephrologist cited “unique challenges for people on dialysis with regard to the way the Medicare hospice benefit is set up, how that disenfranchises patients who are on dialysis.”

Furthermore, existing quality metrics disincentivize palliative dialysis. For concurrent care to be more feasible, interviewees suggested that Medicare should prioritize patient-centered outcome measures like quality of life and patient and family satisfaction.

Interviewees noted that patient diversity must be factored into care delivery. One palliative care physician remarked, “Due to systemic factors, racism, economic discrimination, many of the patients […] may not have the resources to do hospice at home, even.” Interviewees described how rural geography and limited transportation options could impede access. Other considerations to ensure equitable care delivery included primary language spoken at home, health literacy, education level, family caregiver availability, and insurance status.

Interviewees described how evidence for concurrent care may drive policy reform. Clinician support was regarded by interviewees as central to concurrent care success in its early stages. Interviewees also emphasized the need to demonstrate economic feasibility to encourage buy-in from payors for policy reform.

### Concurrent Hospice-Dialysis Program Conceptual Model

Informed by the interview data and feedback from the CAP (Table 3), we developed a conceptual model of the Concurrent Hospice-Dialysis Program, noting that some of the earlier phases will be even more relevant as the Program is expanded to include people up to 6 months before the end of life. Each of the five Program phases refers to a distinct clinical status and its corresponding components of care, detailed in Figure 1. The model provides both a conceptual and practical roadmap, delineating chronological progression through the Program:

1. **Prepare**. The patient’s clinical status is stable; they tolerate dialysis and other medical treatments well. This phase may last months to years. This is an appropriate time to engage patients and families in advance care planning, and discussions about end-of-life care on dialysis.
2. **Decide**. The patient’s clinical status is declining; dialysis becomes more difficult to tolerate and hospitalizations are more frequent. Prognosis is months. Clinicians must identify the decline, plan and initiate goals-of-care conversations, and describe and offer the Program.
3. **Manage**. The patient’s clinical status is stable, and the Program is initiated. Guided by symptoms and goals, they tolerate palliative dialysis. Prognosis is weeks. This phase begins with hospice intake and confirmation of patient goals; patient and clinician education shifts towards anticipatory guidance about dialysis-specific end-of-life symptoms and stages.
4. **Intensive support**. The patient’s clinical status is declining. They find palliative dialysis increasingly difficult to tolerate and may miss treatments. Prognosis is days. Clinicians must communicate decline to patients and families; education focuses on caring for patients who are approaching the end of life.
5. **End of life/bereavement**. The patient is at end of life. Dialysis has stopped because it is not tolerated or no longer aligns with patient goals. Prognosis is hours to days. Focus is solely on end-of-life care: hospice addresses the patient’s physical, psychosocial, and spiritual needs. Bereavement support is offered to families and dialysis teams.

**Table 3:** CAP Activities and Takeaways

CAP 1	CAP 2	CAP 3
**Activities** •Introduction to the Concurrent Hospice-Dialysis Program •What you like, what doesn’t work, what we should think about as we develop the program	**Activities** •Program Current State model •Describe the “target state,” short-term goals to reach target state, and how to achieve those goals	**Activities** •Program Conceptual Model with phases •Identify needs within each phase
**Main takeaways** •Importance of patient- and family-facing communication •Policy issues that need to be addressed—funding, definitional issues, and measurement tools •Importance of care coordination between teams	**Main takeaways** •Patients/families want early guidance and goals-of-care discussions •Care coordination, program descriptions, and patient criteria must be standardized •Policy must allow for funding, providers need incentives, quality metrics ought to be considered	**Main takeaways** •Patients/families need education and support resources •Clinical teams need communication training and interdisciplinary education •Strong guidelines are needed to ensure smooth workflow and care coordination

CAP 4 consisted of review and refinement of takeaways from CAPs 1–3

### Concurrent Hospice-Dialysis Program Resource List

The CAP developed and refined a Resource List for the Program to facilitate effective concurrent care. Resources include written and interactive educational materials, communication tools, workflow processes, order sets, and documentation templates. Table 4 lists resources, grouped according to the need they address.

**Table 4:** Resources Identified by the CAP

Category
**Communication training**
How to have dialysis-focused Advance Care Planning conversations
How to bring up and describe the Program in language that patients and families can process (Including palliative dialysis vs. standard dialysis)
How to bring up bring up goals of care when patient is declining
**Team education**
Hospice and dialysis teams to gain reciprocal understanding of each other’s roles and scope of practice
Multi-modal education for dialysis clinicians to understand philosophy of palliative dialysis and concurrent care (module, interview quotes, group activity)
**Patient/family education and roadmap**
Patient/family brochure on the Program
Patient/family workbook that addresses how to live well on dialysis and prepare for the future •What to expect living with dialysis. What will the journey look like? •What’s important to me? •What are points that one would think about stopping dialysis? (“Benefit-burden guide”) •What to expect if I do palliative dialysis or stop dialysis? •Who’s the team? (designated clinicians to guide patients/families, contact information)
**Patient identification**
Standard criteria to identify patients who may benefit (SQ, hospitalizations, frailty)
Dedicated process to evaluate suitability of identified candidates (monthly quality meeting, care plan)
**Workflow**
Which clinicians are involved and at what stages (a “role” guide)
Standardized referral pathway
Troubleshooting common challenges (number of treatments and what to do if exceeded, what to do if a patient revokes hospice, etc.)
**Documentation and care coordination**
Documentation of goals of care in a standard way to make sure all clinicians follow goals consistently across settings (for instance, incorporate into dialysis care plan)
Communication tool (completed by hospice and dialysis team to provide feedback on the process)
Order set for palliative dialysis (completed by dialysis team with palliative dialysis prescription)
**Team/patient/family support**
How to support patients/families throughout the process (“walk hand-in-hand” phrasing)
End-of-life/bereavement support resources

## DISCUSSION

Participants described key components and considerations for the implementation of the Program, emphasizing the necessity of early goals-of-care conversations, clear Program guidelines, and interdisciplinary collaboration, while acknowledging the payment policy barriers. We developed a conceptual framework that describes five Program phases and the resources required to address specific needs—including written and interactive educational materials, communication tools, workflow processes, order sets, and documentation templates—and identified knowledge gaps.

Our previous findings suggest that the option to continue dialysis alongside hospice may improve symptoms and provide patients control.^16, 18^ Providing concurrent hospice and dialysis care may promote less invasive medical interventions and better align with values and preferences of patients with advanced CKD to focus fully or partially on comfort rather than longevity.^25^Further, although medical management of ESKD without dialysis is increasingly prevalent and feasible, dialysis remains the dominant treatment model for people living with ESKD.^2, 4, 26^ Currently, conversations between patients and clinicians about quality of life occur infrequently, and patients and families often recount a lack of decision-making support from nephrology clinicians when discussing discontinuation of dialysis.^27^ Yet, caregivers whose family members stopped dialysis or received dialysis and hospice concurrently prior to death report higher satisfaction.^28^

To this end, we found that early goals-of-care discussions are critical to concurrent care delivery. Although guidelines recommend models for estimating prognosis in late-stage CKD, baseline understanding of prognosis is often overly optimistic, while more accurate understanding of poor prognosis is associated with higher preference for comfort-focused care.^29, 30^ In fact, lower rates of dialysis regret have been observed among people who have discussed life expectancy with their physician and completed a living will.^31^ Such findings have been operationalized for people living with other serious illness: recognizing the importance of treatment discussions in the context of patient’s values, Medicare requires a goals-of-care discussion before ventricular assist device (VAD) implantation for people living with heart failure.^32^

In terms of policy, Medicare relies on measures of dialysis adequacy (e.g., laboratory values) that often require uncomfortable blood draws, limiting flexibility for dialysis organizations to provide palliative dialysis.^33^ Paired with the Medicare Hospice Benefit that limits disease-directed therapy, these financial incentives drive care delivery, leaving patients who may be interested in palliative dialysis with limited options.^34^ Recent evidence bears this out, as concurrent care is most common for Veterans Affairs-financed hospice compared to Medicare.^35^ Further, Black patients living with ESKD and those living in rural areas disproportionately lack access to hospice and experience intensive care at the end of life in the current model.^36, 37^ Work to improve hospice access is timely: Medicare is testing innovative payment models for concurrent care delivery.^38^ Clinical resources such as our Program may be able to facilitate scalable and equitable implementation of potential policy changes.

### Limitations

Although the Program was implemented at a single site and included a relatively small number of patients, we included clinicians and administrators from diverse geographies and health systems who did not participate in the Program with nuanced perspectives on the feasibility of such programs. Due to the nature of patients’ late-stage disease during participation in the Program, we included patients who did not have direct experience with the Program. Even still, we had limited representation by race, gender, and urban/rural geography, and we may not have identified unique perspectives from a more diverse sample, though we sought diverse family caregiver perspectives, including bereaved family caregivers of Program participants. Future work will intentionally test the Program in more diverse settings in terms of race, ethnicity, and geography.

### Conclusions

We developed a conceptual model for concurrent hospice-dialysis care and corresponding resource list to facilitate concurrent care delivery. The Program must be systematically evaluated via a hybrid implementation-effectiveness trial. Clinical implementation and educational resources can facilitate scalable and equitable dissemination of concurrent care.
